# Rapid development of drug resistance during initial dolutegravir-based antiretroviral therapy of an infant with HIV

**DOI:** 10.4102/sajhivmed.v26i1.1750

**Published:** 2025-10-09

**Authors:** Natasha L. O’Connell, Tara-Lee von Mollendorff, Gert van Zyl, Stephen Korsman, James Nuttall

**Affiliations:** 1 Department of Paediatrics and Child Health, Faculty of Health Sciences, University of Cape Town, Cape Town, South Africa; 2 Division of Medical Virology, Department of Pathology, Faculty of Medicine and Health Sciences, Stellenbosch University, Cape Town, South Africa; 3 National Health Laboratory Service, Tygerberg Business Unit, Cape Town, South Africa; 4 Division of Medical Virology, Department of Pathology, Faculty of Health Sciences, University of Cape Town, Cape Town, South Africa; 5 Health Laboratory Service, Groote Schuur Hospital, Cape Town, South Africa

**Keywords:** children, HIV, drug resistance, dolutegravir, virological failure, perinatal transmission

## Abstract

Dolutegravir resistance is predominantly reported in antiretroviral therapy-experienced individuals. We describe an infant who developed high-level resistance to abacavir, lamivudine, and dolutegravir within 97 days of initiation, despite initial wild-type infection. Causative factors likely include probable sub-therapeutic antiretroviral drug levels, poor tolerance, severe immunocompromise, and a high pre-treatment viral load.

**What this study adds:** The evolution of DTG resistance from a confirmed transmitted wild-type virus in this infant is more rapid than previously described. Identification of risk factors for developing DTG resistance in infants requires further investigation.

## Patient presentation

A 4-month-old female full-term infant, breast- and formula-fed since birth, was diagnosed with HIV when admitted to intensive care for severe pneumonia due to cytomegalovirus (CMV) and *Pneumocystis jirovecii*. Her CD4 count was 153 cells/µL (30%). As per the national guidelines,^1^ no baseline HIV viral load (VL) was done; however, the positive HIV total nucleic acid test, with cycle threshold value of 16.7, suggested a high concentration of either HIV-1-RNA, -DNA, or both. Abacavir/lamivudine/dolutegravir (ABC/3TC/DTG) was initiated along with ganciclovir, followed by valganciclovir for 6 weeks, prednisone, and cotrimoxazole for 21 days, followed by daily cotrimoxazole prophylaxis.

She was discharged after prolonged oxygen treatment and difficulty establishing oral feeds, and was re-admitted within 2 weeks with acute gastroenteritis. An ophthalmology review 3 weeks later showed left eye CMV retinitis; valganciclovir was reinitiated. The mother reported poor feed and medication tolerance.

At 7 months of age (3 months after ART initiation) she was re-admitted with septic shock resulting from *Pseudomonas aeruginosa* bacteraemia. Her HIV VL at that time was > 10 million copies/mL (> 7 log_10_) and CD4 count 41 cells/µL (17%). Despite continued valganciclovir treatment, CMV retinitis progressed to bilateral disease with left macula involvement threatening her vision and requiring regular intravitreal ganciclovir injections.

HIV drug resistance (HIVDR) testing 10 days after re-admission revealed high-level resistance to ABC, 3TC, and DTG. ART was switched to zidovudine/lamivudine/lopinavir/ritonavir (AZT/3TC/LPV/r), < 4 months after starting the primary ART regimen.

Within 3 weeks of initiating AZT/3TC/LPV/r, *Mycobacterium bovis* Bacillus Calmette-Guérin (BCG) disease, with right axillary and chest wall abscesses and pneumonia, was diagnosed on induced sputum. She was treated with rifampicin, isoniazid, ethambutol, and levofloxacin for 9 months, and LPV/r was boosted with additional ritonavir.

Both parents were newly diagnosed with HIV at the time of the infant’s diagnosis. The mother reported having tested HIV negative during a previous pregnancy. Both parents reported being ART-naïve and were started on tenofovir/lamivudine/dolutegravir (TLD), achieving early viral suppression. Alternative mechanisms for HIV infection in the infant, such as blood transfusions, scarification and non-maternal breastmilk exposure, were not reported.

Since DTG resistance is unexpected so soon after starting first-line ART, we aimed to establish whether drug resistance was transmitted from the mother to the child, or developed rapidly in the infant.

## Methods

HIV drug resistance testing of the HIV polymerase (*pol*) gene was performed on ethylenediaminetetraacetic acid blood samples from the mother and repeated for confirmation on the infant 2 months after initiation of AZT/3TC/LPV/r. The father was virologically suppressed on TLD, and HIVDR testing was not performed.

The infant’s VL was 13 355 copies/mL, enabling bulk 3 kb *pol* gene sequencing^2^ covering integrase (int), reverse transcriptase (rt), and protease (pro). The mother’s VL of 63 copies/mL required two separate assays to amplify shorter regions: a published assay to amplify the HIV-1 group M integrase gene,^3^ and a published assay to amplify HIV-1 *gag* p6, protease and reverse transcriptase (p6-pro-rt).^4^ Assays were performed at limiting dilution, yielding eight amplicons for integrase and three for p6-pro-rt.

We also sequenced the original dried blood spots (DBS) collected from the infant at diagnosis to determine whether HIVDR was present at diagnosis.

Sequencing libraries were prepared using Oxford Nanopore Technologies Native Barcoding Kit 96 V14 (SQK-NBD114.96), and sequenced on R10 ONT Flongle flow cells. The Nano-RECall pipeline was used for sequence alignment and correction. Relatedness between infant and maternal sequences was assessed with neighbour joining phylogenetic analysis. Drug resistance interpretation was with the Stanford HIV Drug Resistance Database.

### Ethical considerations

Written consent for the publication of this case report was obtained from the mother and ethical approval from the University of Cape Town (reference number: HREC REF: 322/2025).

## Results

**FIGURE 1:**
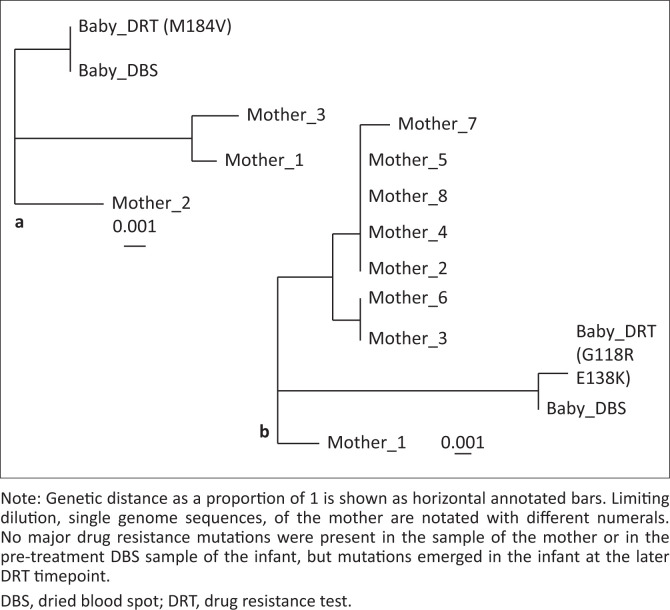
Neighbour-joining phylogenetic trees with major mutations in parenthesis. (a) Protease and reverse transcriptase sequences and (b) integrase sequences.

Neighbour joining phylogenetic analysis revealed high (> 98%) sequence identity between the mother and infant across all HIV enzymes (Figure 1), with similar polymorphisms, corroborating mother-to-child transmission. The infant’s sample from 2 months after switching to AZT/3TC/LPV/r showed no protease inhibitor mutations, a nucleoside reverse transcriptase inhibitor (NRTI) mutation, M184V, and two major DTG-associated mutations, G118R and E138K, which were absent in the mother’s sample and the infant’s original DBS. This supports the rapid acquisition of HIVDR during 97 days of DTG-containing ART.

## Discussion

Dolutegravir-based ART is now the preferred first- and second-line treatment across all populations because of superior tolerability, few drug interactions, and high genetic barrier to resistance. South Africa adopted DTG-based regimens in 2019.^1^

In this case, HIVDR testing revealed several mutations: M184V/I, conferring high-level resistance to 3TC and low-to-intermediate resistance to ABC; G118R, associated with virologic failure on DTG and causing a > 10-fold reduction in DTG susceptibility; and E138K, linked to integrase strand transfer inhibitor use, reducing drug efficacy further with other mutations.^5^

Dolutegravir resistance is rare in treatment-naïve individuals who start treatment on DTG-based ART; however, high baseline VL, severe immunosuppression and sub-therapeutic drug levels are key risk factors.^6^ This infant was virally unsuppressed and severely immunosuppressed after 97 days on ART (VL > 7 log_10_; CD4 count 41 cells/µL). High viral replication increases the chance of mutations, and poor feed tolerance may have led to sub-therapeutic ART levels, further contributing to developing resistance. Advanced HIV disease with severe immunosuppression and active opportunistic infections can be associated with malabsorption and impaired ART absorption.^6^ At this stage the infant was not on rifampicin, which can further reduce DTG drug levels. Zinc was prescribed for 10 days, but details on timing of administration in relation to ART to prevent reduction in DTG levels were not documented.

High-level baseline DTG resistance has been reported in two children who were newly diagnosed with HIV (14 and 18 months of age, respectively).^7,8^ In both cases (R263K in one, and E138K and G118R mutations in the other), the mothers were on DTG-based ART during pregnancy and/or breastfeeding with intermittently unsuppressed VLs resulting from treatment interruptions, but HIVDR testing was not performed on them. Since paired mother–child HIVDR testing at the time of infant diagnosis was not done, HIVDR in these children was unexplained and may have been acquired because of prolonged low-level DTG exposure through breastmilk. As the mothers ultimately had a favourable VL response to DTG treatment, transmitted resistance is unlikely.

This report provides strong evidence of rapidly acquired DTG resistance and, in contrast to previous reports, we were able to show the acquisition of drug resistance between an initial DBS sample and a subsequent sample 3 months later. Additionally, paired maternal-infant HIVDR testing corroborates acquisition of drug resistance after transmission.

## Conclusion

These findings support the rapid acquisition of DTG resistance in an infant treated with ABC/3TC/DTG. Likely contributing factors are the high VL, probable reduced ART drug levels because of poor tolerance, and severe immunosuppression, which favour accelerated viral replication and resistance evolution.
